# Artificial Intelligence-Based Screening System for Diabetic Retinopathy in Primary Care

**DOI:** 10.3390/diagnostics14171992

**Published:** 2024-09-09

**Authors:** Marc Baget-Bernaldiz, Benilde Fontoba-Poveda, Pedro Romero-Aroca, Raul Navarro-Gil, Adriana Hernando-Comerma, Angel Bautista-Perez, Monica Llagostera-Serra, Cristian Morente-Lorenzo, Montse Vizcarro, Alejandra Mira-Puerto

**Affiliations:** 1 Ophthalmology Service, Hospital Universitari Sant Joan, Institut d’Investigació Sanitària Pere Virgili [IISPV], Universitat Rovira i Virgili, 43204 Reus, Spain; romeropere@gmail.com (P.R.-A.); raul_navarro_gil@hotmail.com (R.N.-G.); adriana.hernando.comerma@gmail.com (A.H.-C.); abautistaper@gmail.com (A.B.-P.); monillagostera@hotmail.com (M.L.-S.); cristian_ml87@outlook.com (C.M.-L.); montseviz@hotmail.com (M.V.); a.mira.puerto@gmail.com (A.M.-P.); 2 Responsible for Diabetic Retinopathy Eye Screening Program in Primary Care in Baix Llobregat Barcelona (Spain), Institut d’Investigació Sanitaria Pere Virgili [IISPV], 43204 Reus, Spain; bfontoba@gmail.com

**Keywords:** diabetic retinopathy, artificial intelligence, algorithm, diabetic retinopathy screening, primary care

## Abstract

Background: This study aimed to test an artificial intelligence-based reading system (AIRS) capable of reading retinographies of type 2 diabetic (T2DM) patients and a predictive algorithm (DRPA) that predicts the risk of each patient with T2DM of developing diabetic retinopathy (DR). Methods: We tested the ability of the AIRS to read and classify 15,297 retinal photographs from our database of diabetics and 1200 retinal images taken with Messidor-2 into the different DR categories. We tested the DRPA in a sample of 40,129 T2DM patients. The results obtained by the AIRS and the DRPA were then compared with those provided by four retina specialists regarding sensitivity (S), specificity (SP), positive predictive value (PPV), negative predictive value (NPV), accuracy (ACC), and area under the curve (AUC). Results: The results of testing the AIRS for identifying referral DR (RDR) in our database were ACC = 98.6, S = 96.7, SP = 99.8, PPV = 99.0, NPV = 98.0, and AUC = 0.958, and in Messidor-2 were ACC = 96.78%, S = 94.64%, SP = 99.14%, PPV = 90.54%, NPV = 99.53%, and AUC = 0.918. The results of our DRPA when predicting the presence of any type of DR were ACC = 0.97, S = 0.89, SP = 0.98, PPV = 0.79, NPV = 0.98, and AUC = 0.92. Conclusions: The AIRS performed well when reading and classifying the retinographies of T2DM patients with RDR. The DRPA performed well in predicting the absence of DR based on some clinical variables.

## 1. Introduction

Type 2 diabetes mellitus (T2DM) is a chronic disease associated with insulin resistance that causes hyperglycemia. It mainly affects the brain, heart, kidneys, and eyes through vascular involvement, causing strokes, myocardial infarctions, nephropathy, and diabetic retinopathy (DR).

According to data from the International Diabetes Federation (IDF), the estimated global prevalence of T2DM in 2021 was 536 million people and is expected to affect 783 million people by 2045. However, the prevalence of diabetes varies widely across regions of the world. The prevalence is highest in the Middle East and North Africa, affecting 16.2% of their populations. As for North America and the Caribbean, the prevalence is 14%. On the other hand, Europe and Southeast Asia have the lowest prevalence, estimated at 9.2% and 8.7%, respectively [1]. In Spain, a study conducted by di@bet.es reported that 7.9% of the population is known to have DM2, although its true prevalence is estimated to be double that, at 13.8% [2].

According to IDF, the global prevalence of diabetic retinopathy (DR) was 22.27% in 2020. This means that the number of adults worldwide with DR is 103.12 million and is expected to increase to 160.50 million by 2045. The prevalence of DR is highest in Africa (35.90%), North America (35%), and the Caribbean (33.30%) and is lowest in South and Central America (13.37%) [3].

Diabetic retinopathy is the leading cause of preventable vision loss and blindness in the working-age population, resulting in a major impact in terms of health and economy. Direct costs associated with DR increase as the disease stages progress, with costs being highest in the sight-threatening stages of DR (STDR). In the Singapore study by Zhang et al. [4], the total cost for patients with STDR was 3.79 times that of patients without DR. Similarly, a USA study showed that diabetic patients with STDR had twice the DR-related cost of the milder cases [4]. Finally, a study in Germany found that the ratio of DR-related costs for mild, moderate, and STDR relative to no DR were 1.2, 2.5, and 3.2, respectively [5].

All diabetic patients have the potential to develop DR; therefore, regular screening and early diagnosis are essential and recommended by ophthalmology associations. It can be detected by fundus examination or by retinography, which is more commonly recommended and forms the basis of current DR screening protocols [6,7].

In developed countries, DR screening is most often performed by ophthalmologists, who examine the patients’ retinas under pupil dilation, or through telemedicine, where patients come to the screening center to have a photograph of their retinas taken, which is then read by experts remotely. That said, early detection of DR is difficult in the current screening systems because of such huge demand. In addition, a significant number of diabetic patients who live far away from screening sites fail to attend scheduled visits, and, as a result, fewer than 50% of diabetes patients have regular examinations [8]. 

Despite the effectiveness of teleretinal screening programs, they are not cost-effective because the manual grading requires many specialists to be involved in it [9]. Therefore, an inexpensive, accurate, and automated method for classifying fundus photographs for DR detection in the primary care clinical setting would greatly benefit providers, healthcare systems, and patients.

Over the last decade, several groups have developed artificial intelligence reading systems (AIRSs) capable of reading retinographies, aimed to alleviate the screening for DR. Most AIRSs are deep learning algorithms (DLAs) that provide a binary classification to distinguish patients with more evolved DR types or referable DR (RDR) from those with no or mild DR [10,11]. Currently, existing DLAs have demonstrated similar or even better performance than human experts on several DR classification tasks [10]. Furthermore, these AIRSs appear to be much more cost-effective than manual assessment, which is the current protocol. A study in Scotland showed that the costs of a national DR screening program can be halved by replacing manual grading with automated assessment [12]. Similarly, another UK study reported a cost saving of 21% [13].

To complement that, other research groups have developed DR prediction algorithms (DRPAs) based on clinical variables aimed at evaluating the risk to each diabetic patient of developing sight-threatening DR (STDR) and, thus, personalizing the screening schedules. 

Across the world, primary care center teams are generally not involved in DR screening due to their lack of training and lack of equipment available, but in countries where primary care is better funded, most diabetic patients will visit their primary care center at some time each year for various reasons [14]. Health managers should try to take advantage of that by devising a screening system in which primary care teams are front and center. 

In this article, we tested an AIRS aimed at identifying T2DM patients with RDR (moderate and severe DR) and a DRPA that can estimate the risk of developing DR to each T2DM patient according to nine clinical variables. 

## 2. Overview of the Published Articles

Below is a summary of the most relevant published AIRSs and DRPAs. Regarding AIRSs, the IDX-DR (Digital Diagnostics, Coralville, IA, USA) was the first algorithm to obtain FDA approval in 2018 [15]. It obtained a sensitivity (S) of 96.8% and a specificity (SP) of 59.4% when detecting RDR. It was tested with Messidor-2, but the datasets used for training and validation were not provided.

Another AIRS that has obtained CE Class IIa Mark and FDA approval was EyeArt^®^ (Eyenuk, Woodland Hills, CA, USA), which was independently validated and tested by the UK National Health Service (NHS) [16]. The results showed an S of 91.3% and an SP of 91.1% when detecting DR [17]. EyeArt was tested offline in India using the Remidio Fundus on Phone device to capture fundus images after pupillary dilation. This study achieved remarkable levels of S and SP for the detection of any degree of DR.

Retmarker^®^ (retmarker SA, Taveiro, Portugal) also obtained CE Mark Class IIa approval. It was evaluated using over 100,000 fundus images from 20,258 consecutive NHS patients in the UK. It gave 95.8% S and 63.2% SP when detecting RDR. The datasets used for training and validation were not provided. An interesting feature of Retmarker is its ability to compare images to detect DR progression based on the number of microaneurysms present [18]. Furthermore, Retmarker demonstrated a 48% reduction in workload in DR detection in Portugal. 

RetCAD (Thirona, The Netherlands) is an algorithm trained to detect DR and age macular degeneration. It achieved an S of 86.1% and an SP of 91.6% for RDR disease. It was validated using the MESSIDOR database [19].

The Eye Research Institute and the National University of Singapore developed SELENA+ to detect RDR, glaucoma, and age macular degeneration [20]. They designed the system to operate autonomously or semiautonomously, with human assistance. It was trained and tested on approximately 500,000 retinal images from diverse datasets. In 2019, SELENA+ was approved by the Singapore Health Service Authority to be implemented in the national DR screening program. Compared to 17 human assessors, SELENA+ performed with comparable accuracy and spent significantly less time. 

In China, an AIRS was developed using over 70,000 retinographies from a web-based platform, LabelMe (Guangzhou, China). External validation of this algorithm was performed using over 35,000 images from population-based cohorts of Malaysians, Caucasian Australians, and Indigenous Australians. The S and SP were found to be 92.5% and 98.5%, respectively [21].

ARDA (Automated Retinal Disease Assessment) is an AIRS developed by Verily Life Sciences LLC (South San Francisco, CA, USA) [22]. This algorithm was developed using datasets of approximately 130,000 retinal photographs of patients with DM from the USA, and for the validation process, it used approximately 10,000 retinographies extracted from Messidor-2 and EyePACS datasets. The algorithm demonstrated an S of 96.8%, which was higher than that of the human graders (74%), while the SP was comparable (96–97%).

Finally, a retrospective validation study compared seven different AIRSs for detecting RDR that had been previously validated [23]. The investigators found that most of the algorithms performed no better than human graders. The sensitivities varied widely (51.0–85.9%), although high negative predictive values (82.7–93.7%) were observed. Interestingly, one algorithm was significantly worse than human graders, missing up to a quarter of cases of advanced DR (sensitivity of 72.4% for RDR), a limitation that could potentially lead to vision loss. The characteristics of the main validated AIRSs are shown in Table 1 together with our MIRA algorithm. 

Regarding DRPAs, three main algorithms have been developed that predict the presence of vision-threatening DR. Aspelund et al. [24] based their method on the level of HbA1c, type of diabetes, value of systolic pressure, the current age, and diabetes duration. It was constructed from the diabetic retinopathy database managed at the Ophthalmology Department of Aarhus University Hospital in Denmark. The database was built based on the clinical data of 5199 patients over 20 years, thus allowing the algorithm to be tested prospectively. The model provides a recommended interval for follow-up fundus screening for the presence of sight-threatening vision of between 6 and 60 months. The algorithm has been further tested in other countries, such as Spain, where Soto Pedre [25] studied 508 patients with T2DM. The results showed that 3.1% developed STDR before their subsequent screening visit, with the value of the area under the curve (AUC) being 0.74. Finally, in the UK, Lund [26], using a sample of 9690 DM patients followed for 2 years, reported that the algorithm predicted the onset of DR stages with AUC values of 0.833 for the T2DM patients.

The Scanlon method is based on the age of patients and their levels of HbA1c and cholesterol [27]. This algorithm was validated with 15,877 patients, obtaining an AUC of 0.77 for predicting the development of STDR.

Finally, the model developed by Broadbent [28], also known as the Liverpool Risk Calculation Engine, was built to detect the risk of developing STDR. The statistical analysis of this algorithm found an AUC value of 0.88 in the prediction of STDR, with an S of 0.61 and an SP of 0.93. We should highlight that this is the only report that provided sensitivity and specificity data. This algorithm was tested on a population of DM patients from Liverpool (UK) and was published in the form of an action protocol called the ISDR protocol. Table 2 and Table 3 describe the clinical variables used to construct each DRPA and their performance compared to our algorithm (Retiprogram), respectively.

## 3. Subjects

### 3.1. Setting

In the database of the Catalan public health system (SIDIAP), there are about 600,000 patients registered with DM2. For 265,388 of these cases, we had access to their electronic health records (EHRs) and their retinographies. On the other hand, in our database (Hospital Universitari Sant Joan de Reus, Catalonia, Spain), there are currently 21,087 T2DM patients registered, for whom we have their complete EHR and retinographies.

### 3.2. Datasets

To test the AIRS, we used 17,297 fundus images, of which 16,097 corresponded to 7389 patients in our database who had undergone annual eye screening for DR during the period from 1 February 2017 to 8 September 2023, and the 1200 fundus images that comprise the public Messidor-2 database [29]. Previously, we had used two different retinographies datasets to build and train the AIRS, with a total of 103,815 images: our sample of 15,123 tagged fundus images (different patients from the validation and testing phases) and 88,692 retinal images extracted from EyePACS [30]. Finally, 5000 fundus images from our database were used to validate the AIRS.

We used the EHR of 40,129 patients with type 2 diabetes mellitus from the SIDIAP database for whom we had the nine clinical variables necessary to test the DRPA during the 11-year follow-up period. We had previously used the EHR of a sample of 101,802 patients with type 2 diabetes mellitus extracted from SIDIAP to validate the predictive algorithm.

### 3.3. Design

To test the ability of the AIRS to read and classify fundus images, we compared the DR categories obtained by the algorithm with those DR categories assigned by a panel of retina experts. In addition, to test the performance of the DRPA, we compared the binary classification predicted by the algorithm (presence or absence of any type of DR) with the classification provided by retina specialists.

### 3.4. Inclusion and Exclusion Criteria

#### 3.4.1. Inclusion Criteria

- Type 2 diabetic patients included in the SIDIAP database for whom we had all the clinical and demographic variables necessary to feed the DRPA during the 11-year follow up study period.
- Type 2 diabetic patients from our database and Messidor-2 database with high-quality retinographies to feed the AIRS.

#### 3.4.2. Exclusion Criteria

- Type 1 diabetic patients.
- Gestational diabetes.
- Patients who did not give informed consent.
- Type 2 diabetic patients with incomplete EHR or poor-quality retinographies.

## 4. Materials and Methods

### 4.1. Ethics and Consent

CEIM IISPV (Institut d’Investigació Sanitària Pere Virgili) approved the present study (approval code RetinaReadRisk, protocol version 1. 3 October 2022, Reference number CEIM: 071/2022), which was carried out in accordance with the revised guidelines of the Declaration of Helsinki. All patients included in the study were previously informed about its objective. Once their commitment to participate in the follow-up of the study was obtained, they were asked to sign the informed consent.

### 4.2. The Algorithms

#### 4.2.1. Artificial-Intelligence-Based Reading System

##### Model Construction and Training

Briefly, our baseline model used a 3 × 640 × 640 input image obtained from a minimal preprocessing step, in which only the external background borders were trimmed and later resized to the required input size. The model consists of a convolutional neural network with 7 blocks of 2 layers each that progressively reduce the size of the data until it has a receptive field of 64 × 5 × 5 for feature extraction. Each layer is a stack of a 3 × 3 convolution with stride 1 × 1 and padding 1 × 1, followed by batch normalization and an ReLU activation function. The final vector has a size of 64 values, which is obtained from a 4 × 4 average pooling stage. In the last layer, a linear classifier and a softmax function use these 64 features to calculate the probability of each of the DR levels. For optimization of the parameters of this convolutional neuronal network, the quadratic weighted kappa is used as a loss function, because it is more appropriate for ordinal classification [13]. Details of the architecture can be found in this work [31].

All 88,692 retinographies available from EyePACS were used [24] together with a sample of 15,123 tagged retinographies from our database to build and train the AIRS. There were 81,266 retinal images with no DR, 8771 with mild DR, 14,097 with moderate DR, and 4588 with severe or proliferative DR in the training phase. As the incidence of proliferative DR was low in both datasets, we decided to merge the severe and proliferative into the same category [32].

##### Validation

A total of 5000 retinographies were taken from our DR screening database for validation. The results of the DLA when detecting RDR were sensitivity = 0.998, specificity = 0.958, positive predictive value = 0.711, negative predictive value = 0.929, error type I = 0.032, and error type II = 0.001 [32]. The AIRS MIRA 4.0 software was granted registration code SAFE CREATIVE 2007104712196 on 10 July 2020 at 11:24 UTC.

##### Testing

For testing, we used 15,297 retinographies from our database and all 1200 retinal images from Messidor-2. First, the reading was made by the AIRS, and then a second reading was made by four masked senior retina ophthalmologists. Finally, the agreement between ophthalmologists and the AIRS for detecting RDR was calculated.

##### Diabetes Retinopathy Classification

Both retina specialists and the AIRS used the Messidor-2 classification for grading the retinographies into levels of severity. The presence of fewer than 5 microaneurysms on a retinogram was classified as mild DR. When there were more than 5 but fewer than 15 microaneurysms or fewer than 5 retinal hemorrhages, it was classified as moderate DR. Finally, when there were more than 5 retinal hemorrhages or the presence of new vessels, it was classified as severe DR (Table 4) [29]. 

#### 4.2.2. Diabetic Retinopathy Prediction Algorithm 

##### Model Construction and Training

The DRPA consists of a fuzzy random forest of 100 decision trees and three independent variables in each node. To develop the DRPA, we initially used a sample of 2323 type 2 diabetic patients from our database of whom we had all the medical variables to feed it [33]. Then, we retrained the algorithm with a much bigger sample of 139,658 patients extracted from SIDIAP (System for Research and Development in Primary). The output from the DRPA predicted a binary result, the presence or absence of DR. First, we included 18 variables (current age, age at diagnosis of type 2 DM, sex, body mass index (BMI), diabetes duration, diabetes treatment, smoker status, blood pressure control, diastolic tension rate, systolic tension rate, HbA1c%, creatinine, estimated glomerular filtration rate (eGFR), total cholesterol, LDL-cholesterol, HDL-cholesterol, triglycerides, and microalbuminuria. By statistical analysis, we evaluated these variables, and only 9 results were significant after applying the fuzzy random forest model. Finally, we decided to build the DRPA using these 9 variables: current age, sex, diabetes duration, diabetes treatment, good or bad control of blood pressure (bad control was defined as systolic arterial tension > 140 mm Hg or diastolic arterial tension > 90 mm Hg), HbA1c level, eGFR, the microalbuminuria value, and the BMI.

**Figure 1:**
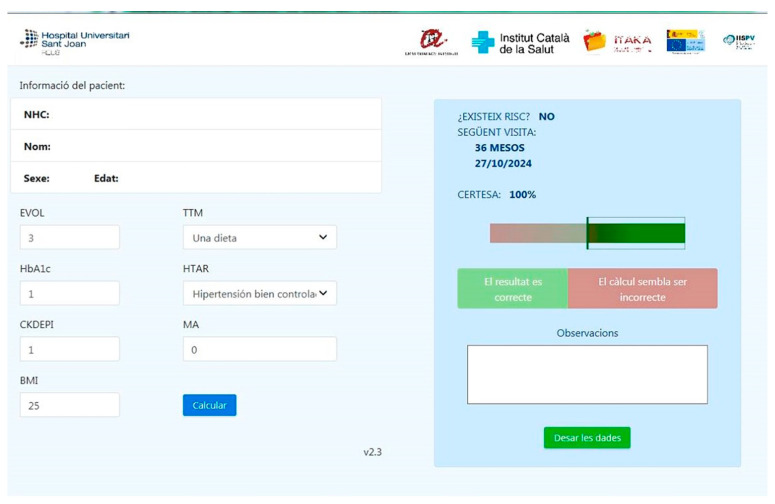
Diabetic retinopathy predicting algorithm interface embedded in the Catalan Health System. In this example, our algorithm (Retiprogram) considered this diabetic patient to be at low risk of developing diabetic retinopathy based on clinical variables and extended the next visit to 36 months.

It estimates the risk to each diabetic patient of having any type of DR according to 9 clinical variables, allowing us to personalize the screening time interval for each patient (Figure 1). Further details are given in our previously published work [34].

##### Validation

Validation was made using the EHR of a sample of 101,802 T2DM patients taken from SIDIAP. Table 5 shows the clinical data of the population with and without DR used to validate the DRPA. The prevalence of DR in the sample was 19,759 patients (19.9%). Microalbuminuria was present in 16,196 patients (14.9%), nephropathy in 1650 patients (1.5%), and dyslipidemia in 26,994 patients (24.9%). There were differences between groups regarding diabetes duration, BMI, glycosylated hemoglobin, microalbuminuria, estimated glomerular filtration rate (eGFR), and arterial hypertension [34].

The results obtained by the DRPA when predicting the presence or absence of DR were accuracy 0.876, sensitivity 84%, specificity 88.5%, positive predictive value 63.8%, and negative predictive value 95.8%. The DRPA Software Retiprogram 4.0 obtained the registration code SAFE CREATIVE 2007144741712 on 14 July 2020 at 6:48 UTC

##### Testing

The DRPA was tested using the electronic medical records of 40,129 patients with type 2 diabetes extracted from SIDIAP. For this study, data from 11 years of follow-up were extracted. First, the DRPA predicted the presence or absence of DR for each diabetic patient based on the 9 clinical variables needed to feed the algorithm. Then, the same four blinded retina specialists provided a definitive result by interpreting their fundus examinations. Finally, we calculated the agreement between ophthalmologists and the DRPA in predicting the presence of DR.

### 4.3. Statistical Methods

Data were analyzed using the SPSS statistical software package (software IBM^®^ SPSS^®^ version 25.0, IBM Corp., Armonk, NY, USA). For qualitative data, frequency and percentage analysis were used in each category and were compared using the χ^2^ test. Quantitative variables are shown as mean ± SD when normally distributed, otherwise the median and interquartile range are shown. In normal distributions, quantitative variables were compared using parametric tests; the Student’s *t*-test was used when it was intended to compare the mean of 2 groups, or the ANOVA analysis was used if there were more than two groups. The receiver operating characteristic (ROC) analysis objectified the variables that best fitted the model prediction. Finally, the variables that were dependent on diabetes duration were assessed through the Cox survival analysis.

We measured the performances of both the AIRS and DRPA using a confusion matrix in which the categories assigned by the algorithms were compared to those given by the retina experts which was considered the ground truth. There were four possible combinations: true positives (TPs) or correct positive assignments, true negatives (TNs) or correct negative assignments, false positives (FPs) or incorrect positive assignments, and false negatives (FNs) or incorrect negative assignments. The performance of the algorithms was evaluated through the following scores: accuracy (ACC), sensitivity (S), specificity (SP), positive predictive value (PPV), negative predictive value (NPV), positive false discovery rate or type 1 error (α), negative false discovery rate or type 2 error (β), and the area under the curve (AUC). *p* < 0.05 was considered statistically significant.

## 5. Results

We show the results obtained by the AIRS when reading and classifying retinal images compared to those provided by retina experts, both in our database and in Messidor-2. Next, we present the results obtained by the DRPA when predicting the presence or absence of DR compared to the categories assigned by retina specialists.

### 5.1. Testing the AIRS in Our Database

The AIRS read 15,297 retinal pictures and only discarded 55 (0.91%) due to poor image quality. Our database contained 12,538 images with no DR (81.9%), 938 with mild DR (6.1%), 1109 with moderate DR (7.2%), and 722 with sight-threatening DR (4.7%).

**Figure 2:**
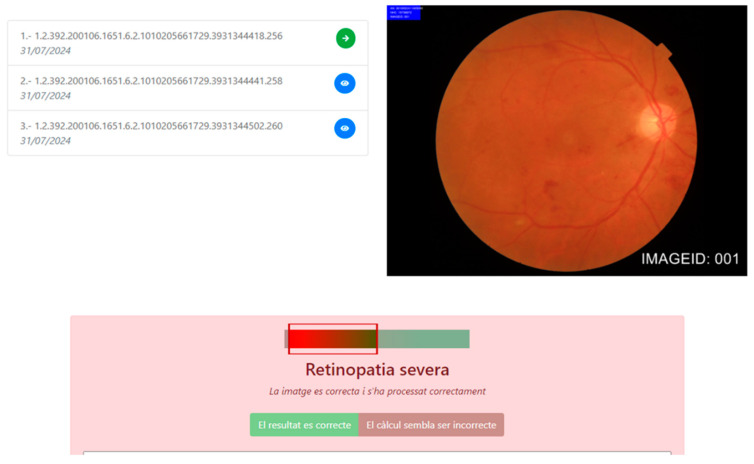
The artificial intelligence-based reading system interface. The algorithm classified this example into severe diabetic retinopathy (Retinopatia severa in Catalan language) due to the presence of multiple retinal hemorrhages.

The algorithm correctly classified 99.9%, 95%, 96%, and 88.7% of those retinographies with no, mild, moderate, and severe DR, respectively. The performance of the AIRS is shown in Table 6. We show an example of the ability of AIRS to classify a retina picture as severe (Figure 2).

Table 7 shows the ability of the AIRS to differentiate those retinographies with nonreferable DR (no DR + mild DR) into those with referable DR (moderate or severe DR). The performance of the algorithm in differentiating those retinographies without RDR from those with RDR was ACC = 98.66, S = 96.7, SP = 99.82, PPV = 99.01, NPV = 98.01, and AUC = 0.958.

The AIRS correctly identified 13,463 (99.5%) and 1706 (96.8%) retinographies with nonreferable and referable DR, respectively. In 64 (3.2%) cases, the AIRS identified RDR when it was not present and misclassified 58 (3.7%) cases as not having RDR when it was present.

### 5.2. Testing the AIRS with Messidor-2

The gradeability test performed by the AIRS on the 1200 retinographies from Messidor-2 found all of them gradable (100%). This was due to the high quality of all the retinal images in that dataset. The AIRS correctly classified 97.6%, 63.3%, 89.3%, and 95.8% of those retinographies contained in the Messidor-2 database with no, mild, moderate, and severe DR, respectively. The algorithm only misclassified 3.4% of these fundus images as having a milder degree of DR than they did (Table 8).

We studied the ability of the AIRS in differentiating those retinographies with nonreferable DR (no DR or mild DR) from those with referable DR (moderate and severe DR). The AIRS identified 373 retinographies that presented referable DR (96.5%) and differentiated them from 809 (99.6%) which had nonreferable DR. The AIRS had only 0.6% and 3.3% of false positives and negatives, respectively (Table 9). 

The algorithm only misclassified 0.16% and 3.3% of nonreferable and referable DR, respectively.

The performance scores when differentiating retina pictures with RDR from those without was S = 94.64%, SP = 99.14%, PPV = 90.54%, NPV = 99.53%, ACC = 96.78%, and AUC = 0.918.

### 5.3. Testing the Diabetes Retinopathy Prediction Algorithm

#### 5.3.1. Clinical and Demographic Data at Baseline

To test the DRPA we had access to 265,388 eligible type 2 diabetic patients from the SIDIAP database, of whom 40,129 patients had the nine clinical variables necessary to feed the DRPA. A total of 22,859 (58%) male patients were included. The mean age was 68.12 ± 10.39 years and the mean duration of diabetes was 9.21 ± 5.51. There were 31,019 (77.3%) patients who controlled their diabetes with oral agents, 4691 (12.6%) with insulin, and 4419 (10.1%) with diet. One-third of them (32%) had arterial hypertension. The mean microalbuminuria and eGFR were 257.3 ± 122.83 and 73.09 ± 15.24, respectively. Table 10 shows the mean values of the clinical and demographic variables of the sample used to test the predictive algorithm for the presence of diabetic retinopathy at baseline.

At the beginning of the study, there were 40,129 T2DM patients, of whom 36,758 had no DR (91.6%) and 3371 had mild DR (8.4%). After 11 years of follow-up, 2924 patients (7.2%) developed some type of DR: 4293 patients (11.5%) showed mild DR, 1398 (3.5%) showed moderate DR, 199 (0.5%) showed severe DR, 164 showed proliferative DR (0.29%), and 241 showed diabetic macular edema (1.05%). In total, 6295 patients (15.6%) presented with some type of DR at the end of the study (Table 11).

#### 5.3.2. Performance of the Predictive Diabetic Retinopathy Algorithm

The algorithm correctly predicted 36,196 and 2795 patients with no and any DR, respectively. In contrast, it predicted that 774 would develop DR when they did not have it (false positives). Finally, it considered that 1090 patients would not develop DR when they did have it (false negatives). S = 0.89, SP = 0.98, PPV = 0.79, NPV = 0.98, and ACC = 0.96. The AUC was 0.92 for any type of DR. 

### 5.4. Patient Journey for the Early Detection of Diabetic Retinopathy in Primary Care

Primary care teams are the health professionals who most frequently care for diabetic patients. This fact should be used to involve primary care teams in eye screening at any opportunistic visit.

**Figure 3:**
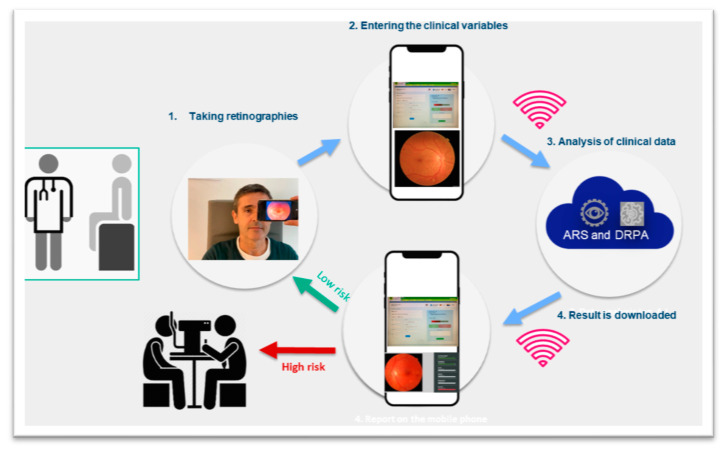
Patient journey according to our eye screening system for the early detection of diabetic retinopathy in primary care. Primary care teams take the retinographies and enter the 9 clinical variables to feed the predictive algorithm. Then, all the data are uploaded to the cloud where the reading and predictive algorithms (the AIRS and the DRPA) evaluate the risk of each patient of developing DR. Only those patients at high risk would be referred to the ophthalmologist. The others would be reevaluated at the primary care level.

Following this scheme, the patient journey is as follows: T2DM patients visit their primary care teams to have their retinographies taken and estimate their risk of developing DR by entering the nine clinical variables to feed DRPA. Then, the clinical data are uploaded to the cloud where they are analyzed by both the AIRS and the DRPA, thus allowing primary care teams to know in real time whether their patients have DR or not and to know their risk of developing it. Only those cases with either DR or high risk of developing it would be referred to the ophthalmologist. The others would be reevaluated according to their risk at different time intervals in the primary care level (Figure 3).

## 6. Discussion

This study was aimed at testing two complementary algorithms for the early detection of DR in T2DM patients. On the one hand, the ability of the artificial-intelligence-based reading system (AIRS) to read and classify the retinographies of patients with DM2 into severity levels using the four standard Messidor-2 categories was tested. On the other hand, the ability of the diabetic retinopathy prediction algorithm (DRPA) to predict the presence or absence of any type of DR based on nine clinical variables was tested. This system was devised to ease the burden of screening for DR and to be applied mainly in primary care.

To build and train the AIRS, 88,692 EyePACS [24] retinal images were used along with a sample of 15,123 labeled retinal images from our database. Then, it was validated using 5000 retinographies from our database and, finally, it was tested using 15,297 retinographies from our database and 1200 retinal images extracted from Messidor-2. 

Our AIRS obtained a sensitivity of 96.7%, a specificity of 99%, a positive predictive value of 99%, a negative predictive value of 99.7%, and an area under the curve of 0.958 when differentiating those photographs without DR or mild DR (nonreferable DR) from those with moderate or severe DR (referable DR) in our diabetic population. When analyzing the Messidor-2 database, the AIRS yielded a sensitivity of 94.6%, a specificity of 99%, a positive predictive value of 90.5%, a negative predictive value of 99.5%, and an area under the curve of 0.918. The false positives shown by the AIRS were 0.08% and 0.86% and the false negatives were 0.1% and 3.3% in our diabetic population and Messidor-2, respectively. Therefore, the AIRS was less accurate when analyzing fundus images extracted from Messidor-2 compared to those in our database. It is noteworthy that our reading algorithm was trained from fundus images taken with a single nonmydriatic fundus camera (TOPCON TRC-NW6S), whereas the fundus images contained in the Messidor-2 database were obtained with several fundus camera models. The difference in pixel definition between the fundus images could explain the better performance of AIRS in our diabetic population compared to Messidor-2.

In 2018, the IDX-DR^®^ reading algorithm was the first to obtain FDA marketing approval for the automatic detection of DR, achieving a sensitivity of 96.8% and a specificity of 59.4% in detecting referable DR. Since then, several groups have built their algorithms, including EyeArt^®^ and Retmarker^®^, which have obtained CE Class IIa marking. EyeArt^®^ showed a sensitivity of 91.3%, a specificity of 91.1%, and an AUC of 0.96 in classifying those retinographs from the UK National Health System with referable DR [17]. Retmarker^®^ also used retinographies from the NHS for its validation, achieving a sensitivity of 73% and a specificity of 85% in detecting any type of DR [35]. It is fair to conclude that our AIRS performed well in both samples in detecting RDR, compared to those that have already been approved by the FDA or that have obtained the CE Class IIa mark. Identifying patients with this level of retinopathy is crucial for referring them to a retina specialist as early as possible and, thus, preventing vision loss and blindness caused by this condition.

The DRPA was tested by conducting a retrospective study of 11 years in a population of 40,129 T2DM patients. It obtained an AUC of 0.92, a sensitivity of 89%, and, more importantly, a specificity of 98% and a negative predictive value of 98%, meaning that the algorithm predicts with sufficient certainty those patients who, due to their clinical characteristics, are not going to develop DR. It allows us to identify patients at low risk of developing DR who would be safe being scanned less frequently, thus freeing up resources for targeting the more urgent cases. When comparing our results with previous studies, it is important to note that only three authors have published work on the development of prediction algorithms: Aspelund [24], Scanlon [27], and Broadbent [28]. 

The model developed by Aspelund was constructed using the EHR from the Diabetic Retinopathy database at the Ophthalmology Department of Aarhus University Hospital (Denmark) over 20 years. They used five clinical variables: the current age, diabetes duration, systolic blood pressure, HbA1c%, and type of diabetes. After feeding the algorithm with the five variables, it recommends a screening time interval ranging from 6 to 60 months for each diabetic patient, depending on their risk of developing DR. The algorithm was tested in Spain [25] and obtained an AUC of 0.74. In the Netherlands, the algorithm was tested by Van der Heijden [36], who obtained an AUC of 0.83, and in the UK, Lund [26] reported an AUC of 0.83 when predicting the onset of DR for T2DM patients.

The model developed by Scanlon was validated using the EHR of T2DM patients from Gloucestershire, UK. This algorithm was constructed from only three independent variables: age, total cholesterol, and HbA1c%. It obtained an AUC of 0.77 for predicting the development of sight-threatening diabetic retinopathy. 

Finally, the model developed by Broadbent was built using seven clinical variables: age at diagnosis, gender, diabetes duration, systolic blood pressure, total cholesterol, HbA1c%, and DR status, to predict the risk of developing sight-threatening diabetic retinopathy. It was tested in the UK and demonstrated a sensitivity, a specificity, and an AUC of 61%, 93%, and 0.88, respectively. It is important to note that all these three algorithms predict only the development of sight-threatening diabetic retinopathy, while our model predicts the presence of any type of DR.

The strengths of the present study are the good performance of our AIRS, not only in our fundus imaging database but also in the public Messidor-2 database. Furthermore, our DRPA not only predicted RDR but any type of DR, and was a very reliable tool to rule out the presence of DR. Limitations of the present study are that we tested our AIRS using fundus images from predominantly Caucasian patients. Therefore, it should be tested on larger samples of patients with greater ethnic diversity. It is worth mentioning that the performance of our AIRS was not as accurate when reading Messidor-2 fundus images compared to those of our population. This is a clear limitation for exporting our algorithm directly to other populations. This fact is probably due to the different image resolutions provided by the different fundus cameras and, therefore, our AIRS must be previously trained and validated in each population that it is intended to be applied on. Another aspect to consider is the different fundus characteristics that different populations far from each other may have, which would force us to validate our algorithm again if we wanted to apply it. Furthermore, the DRPA algorithm was tested using medical records that were collected some years earlier. This fact led us to choose only those medical records from the Catalan Health System Diabetic Patient Database (SIDIAP) in which the data collection process follows a strict protocol and helps to build trust in them.

## 7. Conclusions

The AIRS can be used as a reliable tool for identifying those T2DM patients with referral DR. It allows us to reduce the time needed to read the images and, thus, screen many more patients annually for DR. As a result, those patients with referral DR would be treated early. The DRPA identified those patients who are not going to develop DR, which allows us to extend the intervals of screening for DR with greater security. If both algorithms were applied in primary care, nurses and family physicians would quickly be able to refer those patients at risk of vision loss and, thus, improve their visual prognosis.

## Figures and Tables

**Table 1:** Main validated AI-based algorithms for diabetic retinopathy detection.

Algorithm	Tech	Year	Training Set	Validation Set	Testing Set	DR Classification	Detection	AUC	S (%)	SP (%)
IDX-DR	CNN	2018	N/A	N/A	Messidor-2	FPRC	RDR	N/A	96.8	59.4
EyeArt 2.0	Image analysis technology	2019	EyePACS	N/A	850,908	ICDR	RDR	0.96	91.3	91.1
Retmarker	Recognition of lesions	2011	N/A	N/A	21,514	No RD/RDR	DR/no DR	0.84	95.8	63.2
SELENA+	CNN	2019	ImageNet	N/A	3556	ICDR	RDR	0.95	91.8	98.7
LabelMe	CNN	N/A	71,043	35,201	N/A	N/A	RDR	0.95	92.5	98.5
ARDA	CNN	N/A	130,000	Messidor-2 EyePACS	N/A	N/A	RDR	0.99	87	98.5
MIRA	CNN	2019	Own dataset EyePACS	Own dataset 5000	Own data Messidor-2	Messidor-2	RDR	0.92–0.958	94.6–96.7	99.1–99.8

AUC: area under the curve; S: sensitivity; SP: specificity; CNN: convolutional neural network; N/A: not available; FPRC: Wisconsin Fundus Photograph Reading Center; RDR: referral diabetic retinopathy; ICS; ICDR: International Clinical Diabetic Retinopathy Severity Scale. Any-DR: any type of RD. Our algorithm (MIRA) performed well in detecting RDR compared to the others.

**Table 2:** Algorithms for predicting the presence of diabetic retinopathy.

Algorithm	Age	Sex	DM Duration	DM Treatment	HbA1c	HTA	eGFR	Protein in Urine	BMI	Type DM	Cholesterol
Aspelund	X		X		X	X				X	
Scanlon	X				X						X
Broadbent					X						X
Retiprogram	X	X	X	X	X	X	X	X	X		

Clinical variables used to build each algorithm. Our algorithm (Retiprogram) is based on 9 clinical variables.

**Table 3:** Results obtained by the different diabetic retinopathy predicting algorithms in the testing phase.

Author	Testing Place (Group)	Number of Patients	AUC
Aspelund	Spain (Soto-Pedre)	508	0.74
	UK (Lund)	9690	0.83
Scanlon	Ireland (Smith)	2929	0.77
Broadbent	UK	4460	0.88
Retiprogram	Catalonia (Romero)	40,129	0.92–0.96

Our DRPA (Retiprogram) performed very well compared to the others.

**Table 4:** Diabetic retinopathy (DR) classification according to the criteria used in the Messidor-2 database.

DR Grade	Microaneurysms (μA)	Hemorrhages (H)	Neovascularization
No DR	0	0	0
Mild DR	≤5	0	0
Moderate DR	5 < μA < 15	0 < H < 5	0
Severe DR	μA ≥ 15	H ≥ 5	0/1

μA: microaneurysms; H: hemorrhages; NV; 0 = absence of neovascularization; 1 = presence of neovascularization.

**Table 5:** Clinical and demographic variables used to validate the predictive algorithm for the presence of diabetic retinopathy.

Variable	Without DR	With DR	*p*
Age (y)	68.53 ± 11.06 (30–99)	69.89 ± 9.89 (33–98)	0.684 ^1^
Female (%)	46.67	48.40	0.3802 ^2^
Diabetes duration (y)	7.25 ± 5.20 (0.2–56.99)	11.15 ± 6.90 (0.2–48.87)	<0.001 ^1^
HbA1c (%)	7.22 ± 1.26 (3.5–16.5)	7.82 ± 1.45 (3.8–18.50)	<0.001 ^1^
Microalbuminuria (mg)	34.73 ± 132.65 (0–59.75)	81.08 ± 250.74 (16.24–2999.75)	<0.001 ^1^
Body mass index	30.21 ± 5 (16–38.91)	30.15 ± 5.15 (16.24–40.75)	0.004 ^1^
Creatinine	1.13 ± 0.23 (0.87–1.22)	1.16 ± 0.35 (0.87–1.23)	<0.001 ^1^
eGFR (CKD-EPI)	60.62 ± 7.56 (60.05–69.84)	58.55 ± 9.54 (58.53–69.77)	<0.001 ^1^
Arterial hypertension (%)	33	39	<0.001 ^2^
Cholesterol total	196 ± 41.3 (165–258)	198 ± 43.4 (168–261)	0.883 ^1^
Triglycerides	168 ± 122 (42–298)	168 ± 125 (40–301)	0.386 ^1^

^1^ Chi-squared test; ^2^ Student’s *t*-test. The DR group showed a longer diabetes duration and a higher proportion of patients with arterial hypertension. They also showed higher values of HbA1c, microalbuminuria, BMI, creatinine, and eGFR compared to the others.

**Table 6:** Confusion matrix. Performance of the artificial-intelligence-based reading system (AIRS) in classifying retinographies into each levels of DR severity compared to the classification given by retina experts in our sample of diabetics.

		Predictions Given by the AIRS			
		0	1	2	3
Classification provided by Ophthalmologists	0	12.71	12	0	0
	1	41	892	5	0
	2	9	35	1.065	0
	3	0	17	64	641
	Total	12.621	956	1.129	641

0: No DR; 1: mild DR; 2: moderate DR; 3: severe and proliferative DR. The algorithm correctly classified 91% of retinographies. It only misclassified 12 (0.01%), 46 (4.9%), 44 (3.9%), and 81 (11.2%) of those retinographies with no, mild, moderate, and severe DR, respectively.

**Table 7:** Performance of the reading algorithm when differentiating images with nonreferable DR from those with referable DR in our diabetic database.

		Predictions Given by the AIRS	
		Nonreferable DR	Referable DR
Classification provided by ophthalmologists	Nonreferable DR	13,463	64
	Referable DR	58	1706
		13,521	1770

**Table 8:** Confusion matrix. Performance of the reading algorithm regarding its ability to classify the retinographies extracted from Messidor-2 into levels of severity compared to the classification provided by Messidor-2.

		Predictions Given by the AIRS			
		0	1	2	3
Classification provided by MESSIDOR-2	0	610	35	0	0
	1	13	143	7	0
	2	2	15	116	10
	3	0	4	7	238
	Total	625	197	130	248

0: No DR; 1: mild DR; 2: moderate DR; 3: severe and proliferative DR.

**Table 9:** Confusion matrix. The ability of the AIRS to analyze retinographies with and without referable DR extracted from the Messidor-2 database.

		Predictions Given by the AIRS	
		Nonreferable DR	Referable DR
Classification provided by MESSIDOR-2	Non-referable DR	809	5
	Referable DR	13	373
		822	378

**Table 10:** Variables used to test the predictive algorithm for the presence of diabetic retinopathy.

Independent Variables	Data
Gender Men Women	22,859 (58%) 17,270 (42%)
Age (years)	68.12 ± 10.39 (33–99)
BMI (kg/m^2^)	27.28 ± 5.2 (18–36.81)
Blood pressure control: Normal arterial tension Arterial hypertension	27,288 (68%) 12,841 (32%)
Glycosylated hemoglobin (%) Diabetes duration (years)	7.76 ± 1.61 (4.8–16.6) 9.21 ± 5.51 (0.9–55.98)
Microalbuminuria (mg/24 h) Diabetes treatment	257.3 ± 122.83 (16.23–3155.72)
Diet Oral agents Insulin	4419 (10.1%) 31,019 (77.3%) 4691 (12.6%)
eGFR	73.09 ± 15.24 (60.05–83.25)

BMI: body mass index; eGFR: estimated glomerular filtration rate. In the sample used to test the predictive algorithm, there were more men than women. They were overweight and had reasonably good metabolic control. Most patients were controlled with oral agents. The mean duration of diabetes was less than 10 years. Three-quarters of patients had good blood pressure control and the mean microalbuminuria was almost in the nephrotic range.

**Table 11:** Number and proportion of diabetic patients with any type of DR at baseline and after a 11-year period of follow up.

	Type of DR at Baseline		Type of DR at the End of the Study	
0	36,758	(91.6%)	33,898	(85.5%)
1	3371	(8.4%)	4293	(11.7%)
2			1398	(3.5%)
3			199	(0.5%)
4			164	(0.29%)
5			241	(1.05%)
Patients with any DR			6295	(15.6%)

0: No DR; 1: mild DR; 2: moderate DR; 3: severe; 4: proliferative DR; 5: diabetic macular edema. During the 11-year period of follow-up, 3.3%, 3.5%, 0.5%, and 0.29% of patients developed mild, moderate, severe, and proliferative DR, respectively. In addition, 1% developed diabetic macular edema.

## Data Availability

The database used and analyzed is available from the corresponding author on research request.
